# FAR1-RELATED SEQUENCE (FRS) and FRS-RELATED FACTOR (FRF) Family Proteins in *Arabidopsis* Growth and Development

**DOI:** 10.3389/fpls.2018.00692

**Published:** 2018-06-07

**Authors:** Lin Ma, Gang Li

**Affiliations:** ^1^School of Biological Science and Technology, University of Jinan, Jinan, China; ^2^State Key Laboratory of Crop Biology, College of Life Sciences, Shandong Agricultural University, Tai’an, China

**Keywords:** light signal proteins, FHY3, FAR1, transcription factors, transcription, genetic

## Abstract

Transposable elements make important contributions to adaptation and evolution of their host genomes. The well-characterized transposase-derived transcription factor FAR-RED ELONGATED HYPOCOTYLS3 (FHY3) and its homologue FAR-RED IMPAIRED RESPONSE1 (FAR1) have crucial functions in plant growth and development. In addition, FHY3 and FAR1 are the founding members of the FRS (FAR1-RELATED SEQUENCE) and FRF (FRS-RELATED FACTOR) families, which are conserved among land plants. Although the coding sequences of many putative FRS and FRF orthologs have been found in various clades of angiosperms, their physiological functions remain elusive. Here, we summarize recent progress toward characterizing the molecular mechanisms of FHY3 and FAR1, as well as other FRS-FRF family proteins, examining their roles in regulating plant growth and development. This review also suggests future directions for further functional characterization of other FRS-FRF family proteins in plants.

## Introduction

Light is one of the most important environmental factors affecting plant growth and development. In the past few decades, ongoing research has identified multiple mutants that display defects in their response to various wavelengths of light. In 1993, the *far-red elongated hypocotyls1* (*fhy1*), *fhy2*, and *fhy3* mutants were shown to display an elongated hypocotyl in far-red light but not in white light (Whitelam et al., 1993). FHY1 and its homolog FHL (FHY1-LIKE) interact with phytochrome A (phyA) and are required for phyA translocation from the cytoplasm to the nucleus after exposure to far-red light (Desnos et al., 2001; Hiltbrunner et al., 2005; Zhou et al., 2005). *FHY2* encodes a phyA that specifically responds to far-red light (Whitelam et al., 1993).

*FHY3* and its homolog *FAR1* encode transposase-derived transcription factors (Hudson et al., 1999; Wang and Deng, 2002; Lin et al., 2007). Although FHY3 and FAR1 were derived from transposases, they have evolved diverse and powerful physiological functions in adaptation and domestication. Recent studies have demonstrated that FHY3 and FAR1 play multiple roles in a wide range of cellular processes, including light signal transduction, photomorphogenesis (Wang and Deng, 2002; Lin et al., 2007), circadian clock and flowering time regulation (Li et al., 2011), shoot meristem and floral development (Li et al., 2016), chloroplast division (Ouyang et al., 2011), chlorophyll biosynthesis (Tang et al., 2012), starch synthesis (Ma et al., 2017), abscisic acid responses (Tang et al., 2013), oxidative stress responses (Ma et al., 2016), plant immunity (Wang et al., 2016), and the low-phosphate response (Liu Y. et al., 2017), indicating that FHY3 and FAR1 are crucial for plant growth and development (**Table 1**). In addition, 12 FAR1-RELATED SEQUENCE (FRS) and four FRS-RELATED FACTOR (FRF) family proteins were identified in *Arabidopsis thaliana* (Lin and Wang, 2004; Aguilar-Martinez et al., 2015). Besides FHY3 and FAR1, the physiological and molecular mechanisms of most FRS and FRF family proteins remain largely unknown in plants.

**Table 1:** The physiological functions of FRS-FRF family proteins in *Arabidopsis*.

Cellular process	Targets	Transcriptional regulation	Reference
Light signal transduction	*FHY1/FHL* *COP1*	FHY3/FAR1 (+), HY5 (-) FHY3/FAR1 (+), HY5 (+)	Lin et al., 2007; Li et al., 2010; Huang et al., 2012
Chloroplast division	*ARC5*	FHY3/FAR1(+), FRS4 (+)	Ouyang et al., 2011; Gao et al., 2013
Chlorophyll biosynthesis Immunity response	*HEMB1*	FHY3/FAR1 (+), PIF1(-)	Tang et al., 2012; Wang et al., 2016
Myo-inositol synthesis Oxidative response	*MIPS1/MIPS2*	FHY3/FAR1 (+)	Ma et al., 2016
Starch synthesis	*ISA2*	FHY3/FAR1 (+)	Ma et al., 2017
Circadian clock Flowering time	*ELF4*	FHY3/FAR1/HY5 (+), CCA1/LHY (-)	Li et al., 2011
Diurnal growth Flowering time regulation	*PIF4 Gl*	FRS7 (-), FRS12 (-)	Ritter et al., 2017
Floral development	*CLV3* *SEP2* *STM*	FHY3 (-) FHY3 (+) FRF1 (?)	Li et al., 2016; Aguilar-Martinez et al., 2015
Drought stress ABA response	*ABI5*	FHY3/FAR1 (+)	Tang et al., 2013
Low phosphate response	*PHR1*	FHY3/FAR1/EIN3 (+), HY5 (-)	Liu Y. et al., 2017

*+, positive regulation; -, negative regulation.*

### Protein Structures

Multiple FRS family members, including FHY3 and FAR1, are transcription factors derived from *Mutator-like element* (*MULE*) transposases. Transposases are usually encode by transposable elements and are responsible for cutting and pasting the transposable elements from their original sites to new sites in the chromosome (Joly-Lopez and Bureau, 2014). An analysis of transposase protein structures revealed the presence of an N-terminal DNA-binding domain and a C-terminal catalytic domain. The N-terminal DNA-binding domains recognize specific DNA motifs in the terminal inverted repeats of the transposon and the C-terminal catalytic domains cleave the double-stranded DNA and insert the transferable element into a new genomic location (Feschotte et al., 2002; Makarova et al., 2002).

**FIGURE 1:**
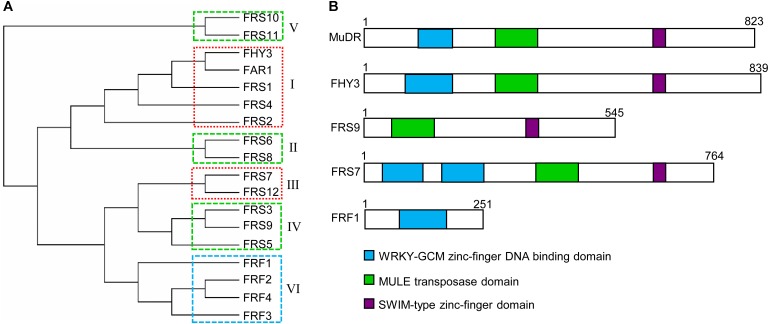
Phylogenetic and protein domain structures of the FRS-FRF family in *Arabidopsis*. **(A)** Multiple alignment of FRS and FRF family proteins were performed using MAFFT (V6.864, http://www.genome.jp/tools-bin/mafft). Phylogenetic analysis was generated using the neighbor-joining method (PHYLYP, V3.66) and displayed with MEGA (v7.0). FRS-FRF family proteins were classified into six subgroups (I–VI) based on their protein structures. The boxes with dashed lines indicate FRS-FRF members in the same clade that might form homodimers or heterodimers to coordinately regulate the transcription of their target genes. **(B)** The conserved protein domains in select FRS-FRF family proteins are shown.

Most members of all FRS subgroups have an N-terminal C2H2 zinc-finger domain (also called a FAR1 DNA-binding domain), a central putative transposase domain similar to *MULE* transposases, and a C-terminal SWIM (SWI2/SNF2 and MuDR transposases) zinc-finger domain (Lin and Wang, 2004; Lin et al., 2007) (**Figure 1B**). Putative nuclear localization signal (NLS) motifs have been identified in most members of the FRS family, including FHY3, FAR1, and FRS2, but not FRS1, FRS8, and FRS9 (Lin and Wang, 2004). The N-terminal FAR1 DNA-binding domain is a type of C2H2 zinc-finger domain from the WRKY-Glial Cell Missing1 (WRKY-GCM1) superfamily, which bind to specific *cis*-elements in the promoter regions of diverse targets (Makarova et al., 2002; Lin et al., 2007).

The putative core transposase domains of FRS family proteins share significant sequence similarity with the transposase domain of MuDR family transposases from elements such as Jittery and MuDRA of maize, and LOM1 of rice (Lin et al., 2007). The transposase activity of FHY3, FAR1, and other FRS family proteins was most likely lost in *Arabidopsis thaliana* despite the high sequence similarity between their core transposase domains and *MULE* transposases (Hudson et al., 2003). By contrast, in maize, the terminal inverted repeats of a putative *FRS5-LIKE* gene were identified as an active transposon inserted in the coding region of *ZmTOM1* in a mutant allele of *yellow striped 3* (*ys3*). This suggests that the transposase activity of the core transposase domain of FRS family proteins might have been retained in maize (Chan-Rodriguez and Walker, 2018).

Mutations of the evolutionarily conserved amino acids in the core transposase domain or C-terminal SWIM domain failed to activate the expression of their target genes, indicating that these two domains are essential for the transcriptional activity, although the underlying molecular mechanism for this regulation remains unclear (Lin et al., 2007). Interestingly, ectopic overexpression of either the N-terminal or C-terminal domains of FHY3 in a wild-type background results in a long-hypocotyl phenotype, but only overexpression of the C-terminal of FHY3 results in a completely etiolated phenotype under far-red light, indistinguishable from the phenotype of *phyA* null mutants. These results indicated that overexpression of C-terminal fragments impairs the function of endogenous FHY3, and also produces a dosage-dependent dominant-negative effect on phyA signaling (Wang and Deng, 2002). It should be noted that FHY3 has been identified as associating with phyA under FR, which suggested that FHY3 might interact with phyA directly (Saijo et al., 2008). In addition, the FHY3 DNA-binding domain is located in the N-terminal, which directly binds to *FHY1* and *FHL* promoters and promotes their gene expression, thus indirectly affecting the translocation of phyA into the nucleus (Lin et al., 2007).

### Classification of FRS-FRF Family Proteins

Multiple sequence alignment and conserved protein motif analyses revealed that *Arabidopsis* FRS and FRF family proteins can be divided into 6 subgroups (**Figure 1A**), consistent with previous studies (Lin et al., 2007; Aguilar-Martinez et al., 2015; Joly-Lopez et al., 2016). These are: subgroup 1 (FHY3, FAR1, FRS1, FRS2, and FRS4), subgroup 2 (FRS6 and FRS8), subgroup 3 (FRS7 and FRS12), subgroup 4 (FRS3, FRS5 and FRS9), subgroup 5 (FRS10 and FRS11, which have unknown functions), and subgroup 6 (FRF1, FRF2, FRF3, and FRF4). Generally, most FRS-FRF family proteins have a DNA-binding domain in their N-terminal regions. As exceptions, in subgroup 3, FRS7 and FRS12 have two DNA-binding domains, and FRS9 of subgroup 4 has no DNA-binding domain in the N-terminal (**Figure 1B**).

In subgroup 6, FRF1–FRF4 have been considered as truncated FRS family proteins since they only contain the FAR1 DNA-binding domain but not the putative core transposase and C-terminal SWIM domains (Aguilar-Martinez et al., 2015). It has been suggested that FRFs might compete with FRSs for the DNA-binding sites of their targets, or regulate the transcription of targets by interacting with other transcription factors; however, determining the underlying molecular mechanism requires further functional studies (Aguilar-Martinez et al., 2015).

### DNA-Binding Activity

The representative FRS family proteins FHY3 and FAR1 bind the FHY3/FAR1-binding site (FBS, CACGCGC) *cis*-elements that reside in the promoter regions of various target genes, including *FHY1*, *FHL*, *EARLY-FLOWERING4* (*ELF4*), *ACCUMULATION AND REPLICATION OF CHLOROPLASTS5* (*ARC5*), *HEMB1*, *CONSTITUTIVE PHOTOMORPHOGENIC1* (*COP1*), *ABA-INSENSITIVE5* (*ABI5*), *CLAVATA3* (*CLV3*), *SEPALLATA2* (*SEP2*), *myo-INOSITOL-1-PHOSPHATE SYNTHASE* (*MIPS1*), *ISOAMYLASE2* (*ISA2*), and *PHOSPHATE STARVATION RESPONSE1*(*PHR1*) (Lin et al., 2007; Li et al., 2011, 2016; Ouyang et al., 2011; Huang et al., 2012; Stirnberg et al., 2012; Tang et al., 2012, 2013; Gao et al., 2013; Wang and Wang, 2015a; Ma et al., 2016, 2017; Liu Y. et al., 2017). Genome-wide chromatin immunoprecipitation-sequencing (ChIP-seq) or DNA affinity purification-sequencing (DAP-seq) analyses have confirmed that FHY3 and FAR1 specifically bind to FBS *cis*-elements in the promoter regions of over 1000 genes in *Arabidopsis* (Ouyang et al., 2011; Li et al., 2016; O’Malley et al., 2016). A recent study revealed that FRS4/CPD25, another member of subgroup 1, could also bind to FBS or FBL (FBS-Like) *cis*-elements in the *ARC5* promoter (Gao et al., 2013). When the essential amino acid in the DNA-binding domain was mutated in FHY3, the mutant protein failed to bind to the FBS *cis*-elements in the *FHY1* and *ARC5* promoters, which indicates that the N-terminal FAR1 DNA-binding domain is responsible for binding to DNA (Lin et al., 2007; Gao et al., 2013).

Although both FRS6 and FRS8 of subgroup 2 have been shown to play a role in flowering time regulation, the *cis*-elements they recognize and the genes they regulate are unknown (Lin and Wang, 2004). FRS6 and FRS8 may recognize the same *cis*-elements in the promoter region of their various targets, since they contain the same essential amino acid in their N-terminal domains. Tandem chromatin affinity purification followed by next-generation sequencing with the subgroup 3 member FRS12 demonstrated that it specifically recognizes FRS12-BOX *cis*-elements (FRB1, TGTGTG; FRB2, TATATATATATATATATAT; FRB3, TATACATA) in the *PIF4*, *GI*, and *PIL1* promoters (Ritter et al., 2017). However, it is still unclear whether subgroup 4 member FRS9 can associate with DNA since it does not have a N-terminal FAR1 DNA-binding domain, even though it contains the putative core transposase and C-terminal SWIM zinc-finger domains. FRS9 might interact with other FRS members to form heterodimers in the nucleus to regulate the transcription of target genes in various cellular processes.

FRF1 from subgroup 6 can bind to the large RB-box in the *SHOOT MERISTEMLESS* promoter (Aguilar-Martinez et al., 2015), however, the specific *cis*-elements and nucleotide sequences required for FRF1 binding to this region have not been identified. There is still no information available on the *cis*-elements recognized by subgroup 5 FRS-FRF members, since FRS10 and FRS11 have not been functionally characterized.

## Physiological Functions of FRS-FRF Family Proteins

### Light Signal Transduction and Photomorphogenesis

Several types of photoreceptors have evolved in plants to perceive environmental light signals. In *Arabidopsis*, phytochromes (phyA and phyB) primarily absorb red or far-red light; cryptochromes (cry1 and cry2) and phototropins (phot1 and phot2) perceive blue and ultraviolet A (UV-A) light; and UVR8 specific perceive UV-B light (Wang and Wang, 2015b). A forward genetic screen identified *FHY3* and *FAR1* as key components in far-red light signal transduction (Whitelam et al., 1993; Hudson et al., 1999; Wang and Deng, 2002). FHY3 and FAR1 form homodimers or heterodimers to directly bind to promoters and activate transcription of *FHY1* and *FHL*, which encode two key regulators of phyA translocation into the nucleus under far-red light. Therefore, FHY3 and FAR1 indirectly affect phyA nuclear translocation (Lin et al., 2007). It should be noted that FHY3 and FAR1 affect phyA nuclear translocation indirectly and in association with phyA, thus playing a role in protecting insufficiently phosphorylated phyA from proteosomal degradation mediated by the COP1 E3 ligase (Saijo et al., 2008). Interestingly, the transcript levels of both *FHY3* and *FAR1* are repressed by far-red light and in phyA-dependent manner by an unknown mechanism (Wang and Deng, 2002; Lin et al., 2007). In addition, ELONGATED HYPOCOTYL (HY5), a well-characterized bZIP transcription factor, physically interacts with both FHY3 and FAR1 directly, and antagonizes their transcriptional activation on *FHY1* and *FHL*, thus affecting far-red light signal transduction (Li et al., 2010).

FHY3 and FAR1 are also involved in UV-B light signal transduction, where they act by directly binding to the promoter of *COP1* and activating its transcription in response to UV-B (Huang et al., 2012). Although FRS4 recognizes the same *cis*-elements as FHY3, it has not been found to be involved in far-red light signal transduction (Gao et al., 2013). Suppression of *FRS9* transcription resulted in a short hypocotyl under red light, but not far-red, or blue light conditions; this observation suggested that FRS9 is also involved in light signal transduction (Lin and Wang, 2004). However, the underlying molecular mechanism is still unclear.

### Chloroplast Division

In higher plants, chloroplasts are the major site for photosynthesis and for the biosynthesis of numerous important components. FRS family proteins affect chloroplast function by regulating chloroplast biogenesis, chlorophyll biosynthesis, and starch synthesis (Ouyang et al., 2011; Tang et al., 2012; Gao et al., 2013; Ma et al., 2017). Chloroplast division determines the number of chloroplasts in each cell and plays an important role in cell expansion, division, and retrograde signal transduction from the chloroplast to the cytoplasm and the nucleus (Gao et al., 2003; Maple and Moller, 2007; Osteryoung and Pyke, 2014). Although many critical components involved in chloroplast division such as ARC5, ARC6, PLASTID DIVISION1 (PDV1), and PDV2, have been identified, the regulation of chloroplast division is still mysterious (Miyagishima et al., 2006; Glynn et al., 2008).

*ARC5* encodes a dynamin-related protein that is essential for chloroplast division, since disruption of *ARC5* results in enlarged, dumbbell-shaped chloroplasts (Gao et al., 2003). Recent studies unexpectedly revealed that FRS family proteins have important roles in the transcriptional regulation of *ARC5* (Ouyang et al., 2011; Gao et al., 2013; Chang et al., 2015). Disruption of either *FHY3*, *FAR1*, or *FRS4* results in enlarged, dumbbell-shaped chloroplasts, while constitutive overexpression of *ARC5* in these mutants restores normal chloroplast morphology, demonstrating that these three FBSs are all required for the transcriptional activation of *ARC5*, albeit by distinct molecular mechanisms (Ouyang et al., 2011; Gao et al., 2013). FHY3 and FAR1 form homodimers or heterodimers, which bind directly to the FBS and FBS-Like (FBL) *cis*-elements in the *ARC5* promoter to activate its expression (Ouyang et al., 2011). Another possibility is that FRS4 might regulate the transcription of *ARC5* by forming heterodimers with FHY3 in the nucleus, since FRS4 alone does not have the ability to regulate transcription (Gao et al., 2013).

### Chlorophyll Biosynthesis

Chlorophyll biosynthesis is strictly regulated by environmental light-dark cycles through a series of enzymatic reactions (Kobayashi and Masuda, 2016; Larkin, 2016). In the dark, chlorophyll biosynthesis is terminated by the accumulation of the intermediate metabolite protochlorophyllide (Pchlide). In the light, Pchlide is rapidly converted to chlorophyllide and ultimately chlorophyll (Larkin, 2016; Liu X. et al., 2017). The excessive accumulation of Pchlide in plants triggers the production of reactive oxygen species (ROS), which cause cell death and photobleaching (Larkin, 2016; Liu X. et al., 2017).

The etiolated seedlings of *fhy3* and *fhy3 far1* mutants greening rapidly after transferred to light, due to with less Pchlide accumulation by the end of the night, which reduces the production of ROS and increases plant survival and fitness after light exposure (Tang et al., 2012). Furthermore, FHY3 and FAR1 directly bind to the *HEMB1* promoter to activate its transcription. *HEMB1* encodes a 5-aminolevulinic acid dehydratase (ALAD) that catalyzes the synthesis of porphobilinogen (PBG) from 5-aminolevulinic acid (ALA). Thus, FHY3 and FAR1 both act as positive regulators of chlorophyll biosynthesis (Tang et al., 2012). Besides *HEMB1*, the expression level of other chlorophyll biosynthesis-related genes including *HEMA1*, *HEMA3*, *FERROCHELATASE 2* (*FC2*), and *HEME OXYGENASE 1* (*HO1*) are also significantly affected in the *fhy3* and *fhy3 far1* mutants, which indicates that FHY3 and FAR1 might regulate chlorophyll biosynthesis through transcriptional regulation of multiple diverse genes (McCormac and Terry, 2002; Tang et al., 2012).

PHYTOCHROME-INTERACTING FACTOR1 (PIF1) is a negative regulator of photomorphogenesis and chlorophyll biosynthesis that physically interacts with FHY3 and partially inhibits transcription of *FHY3*, which in turn regulates the expression of *HEMB1* and chlorophyll biosynthesis (Tang et al., 2012). In addition, ETHYLENE-INSENSITIVE 3 (EIN3) and EIN3-LIKE 1 (EIL1), two transcription factors of the ethylene signaling pathway, directly bind to the promoters and activate the transcription of *PROTOCHLOROPHYLLIDE OXIDOREDUCTASE A* (*PORA*) and *PORB*, which are also essential in chlorophyll biosynthesis (Zhong et al., 2009). It is worthwhile to note that EIN3/EIL1 physically interacts with FHY3/FAR1 and PIF1 (Tang et al., 2012; Liu Y. et al., 2017); however, how these transcription factors work together to coordinate transcriptional regulation of chlorophyll biosynthesis-related genes in early seedling development requires further investigation.

### Starch Synthesis and Starch Granule Formation

In the daytime, sugar produced by photosynthesis in the leaves provides the energy to maintain all kinds of metabolic activities, and any unused sugar is stored for the short term as starch (Streb and Zeeman, 2012). In the night, the transient starch in plant leaves is degraded into sugar to maintain plant growth and metabolism (Streb and Zeeman, 2012). Starch metabolism is regulated by the environmental light-dark cycle and endogenous sugar content, yet little is known about the underlying molecular mechanism (Smith et al., 2005; Graf and Smith, 2011).

A recent study revealed that FHY3 and FAR1 are crucial for starch synthesis but not turnover (Ma et al., 2017). Disruption of *FHY3* and *FAR1* resulted in a decrease in starch content and an increase in water-soluble polysaccharide content in the leaf at end of a light period. In addition, the highly ordered starch granule structure was dramatically disrupted in *fhy3* and *fhy3 far1* mutants, a phenotype that is very similar to that of *isoamylase 2* (*isa2*) mutant plants (Ma et al., 2017). *Arabidopsis ISA1* and *ISA2* encode isoamylase-type debranching enzymes that are essential for starch granule biosynthesis (Delatte et al., 2005; Wattebled et al., 2005). Further investigation showed that FHY3 and FAR1 regulate starch synthesis through transcriptional activation of *ISA2* thus mediate light-induced regulation of starch synthesis during the day (Ma et al., 2017).

### The Circadian Clock and Flowering Time Regulation

Many biological processes have an endogenous oscillation of about 24 h, aligning with the day-night cycle; this oscillation is driven by the endogenous circadian clock (McClung et al., 2002; Doherty and Kay, 2010; Oakenfull and Davis, 2017). In plants, environmental light signals are among the most important factors affecting the entrainment of the circadian clock (Devlin and Kay, 2001; McClung et al., 2002). Multiple components governing light signal transduction, including phyA, phyB, cry1, and cry2 are involved in light entrainment of the circadian clock in *Arabidopsis* (Devlin and Kay, 2001; Harmer, 2009). FHY3 was originally identified for its role in gating the red light signal for clock resetting (Allen et al., 2006). Disruption of *FHY3* causes arrhythmicity of *CAB2* expression under continuous red light but not under continuous blue light (Allen et al., 2006). Further studies revealed that the expression levels of multiple circadian clock-related genes, including *CIRCADIAN CLOCK ASSOCIATED1* (*CCA1*), *LATE ELONGATED HYPOCOTYL* (*LHY*), and *ELF4*, are significantly altered in *fhy3*, *far1*, and *fhy3 far1* mutants. Therefore, FHY3 and FAR1 were proposed to be part of the light input pathway for the circadian clock (Li et al., 2011).

FHY3 and FAR1 directly bind to FBS *cis*-elements in the promoter of the central clock gene *ELF4* to activate its transcription and promote flowering. HY5 also directly binds to the *ELF4* promoter to induce its expression. CCA1 and LHY, two MYB type transcription factors, directly bind to the evening elements (EE) in the *ELF4* promoter to repress its expression at dawn. Furthermore, CCA1 and LHY physically interact with the transcriptional activators FHY3, FAR1, and HY5, to suppress their activation of *ELF4* transcription during the day (Li et al., 2011). FHY3 and FAR1 buffer the transcript level of *ELF4* in the evening in a red light-dependent manner, and act downstream of the light-stable phytochromes phyB, phyD, and phyE (Siddiqui et al., 2016). FHY3 was found to associate with phyA *in vivo* (Saijo et al., 2008). These observations encourage further investigations that aim to identify other phytochromes or photoreceptors that FHY3 associates with *in vivo*.

Multiple FRS family members, including FHY3, FAR1, FRS6, FRS7, FRS8, and FRS12, negatively regulate flowering time by regulating the transcription of a diverse set of genes (Lin and Wang, 2004; Li et al., 2011; Ritter et al., 2017). FHY3 and FAR1 negatively regulate flowering time by activating the transcription of *ELF4*, with FHY3 playing a primary role (Li et al., 2011). FRS7 and its paralog FRS12 negatively regulate flowering time by repressing the transcription of *GIGANTEA*, and FRS7 plays a primary role (Ritter et al., 2017). Since mutating *FRS7* or *FRS12* has no effect on the expression of the central circadian clock genes such as *LHY* and *TOC1*, the FRS7–FRS12 complex might regulate plant growth and development by affecting the output of the circadian clock (Ritter et al., 2017). FRS6 and FRS8 also negatively regulate flowering time, as the disruption of *FRS6* and *FRS8* results in early flowering. However, the downstream targets and underlying molecular mechanism remain unclear (Lin and Wang, 2004).

### Shoot Apical Meristem and Floral Development

In addition to playing multiple roles in early seedling development and flowering time regulation, FHY3 also participates in reproduction in *Arabidopsis*. Genetic evidence has revealed that FHY3 can promote shoot branching and is necessary for shoot meristem determinacy and maintenance (Stirnberg et al., 2012; Li et al., 2016). A genetic screen for suppressors of the highly branched mutant *max2-1* (*more axillary branching2-1*) identified three recessive alleles at the *FHY3* locus, indicating that FHY3 promotes shoot branching (Stirnberg et al., 2012). Mutating *FAR1* also slightly suppressed the highly branched *max2-1* phenotype, but mutating *FHY1* or *PHYA* did not. In addition, a *fhy3-12* mutant displayed reduced axillary bud activity in the strigolactone-deficient mutant backgrounds, *max2-1* and *max4-1*, and cytokinin-overproducing mutant *amp1* (*altered meristem programme1*), but not in the auxin-related *axr1-3* (*auxin resistant1-3*) mutant background. This suggests that FHY3 might be involved in attenuating the auxin-regulated inhibition of bud outgrowth (Stirnberg et al., 2012). However, the molecular mechanism for FHY3-dependent activation of shoot branching requires further investigation.

In a genetic screen for second-site suppressors of *ag-10* (*agamous*-10), FHY3 was also identified as an enhancer of floral meristem determinacy (Li et al., 2016). The mutants alleles of *fhy3-27*, *fhy3-39*, *fhy3-46*, and *fhy3-68* display small petals, sterile anthers, and very short, bulged siliques, which suggests that *FHY3* is necessary for seed reproduction (Li et al., 2016). A combination of ChIP-seq and RNA-seq identified hundreds of direct FHY3 targets that are involved in floral development. Interestingly, FHY3 directly represses the transcription of *CLV3*, but activates the transcription of *SEP2* to ultimately promote floral meristem formation in the shoot apical meristem (Li et al., 2016). In addition, FHY3 might act as a transcriptional repressor of shoot apical meristem and floral meristem development, which is distinct from its roles as a transcriptional activator in light signaling, or light entrainment of the circadian clock during seedling development (Wang and Wang, 2015a; Li et al., 2016).

### Oxidative Stress, Plant Immunity, and Cell Death

FHY3 and FAR1 also negatively regulate ROS accumulation and oxidative stress-induced cell death (Stirnberg et al., 2012; Ma et al., 2016; Wang et al., 2016). The adult *fhy3 far1* mutant plants had slow, stunted growth, accumulated ROS, and displayed severe cell death under short-day or extended darkness conditions (Stirnberg et al., 2012; Ma et al., 2016; Wang et al., 2016). This cell death phenotype can be rescued by overexpressing *SA-3-hydroxylase* (*S3H*) to reduce accumulation of salicylic acid (SA), or by crossing *fhy3 far1* plants with either SA metabolism mutants or signal transduction-related mutants (including *pad4*, *eds1*, *sid2* and *NahG*), which suggests that the cell death phenotype in *fhy3 far1* mutants is largely dependent on the accumulation of SA (Ma et al., 2016; Wang et al., 2016).

Interestingly, loss of *FHY3* and *FAR1* function enhances the expression of defense-responsive genes, thus increasing resistance to *Pseudomonas syringae* bacteria, which indicates that FHY3 and FAR1 are also involved in modulating plant immunity (Wang et al., 2016). Overexpression of *HEMB1*, one of the chlorophyll biosynthesis genes regulated by FHY3 and FAR1, also rescues the cell death phenotype in *fhy3 far1* mutants. However, reducing the expression of *HEMB1* increases the expression of defense-response genes and results in a lesion-mimic phenotype. This genetic and molecular evidence demonstrates that FHY3 and FAR1 negatively modulate plant immunity and cell death, possibly by interfering with biosynthesis of chlorophyll and SA signaling (Wang et al., 2016).

In addition, FHY3 and FAR1 suppress the accumulation of ROS and oxidative-stress-induced cell death partially through positively regulating myo-inositol biosynthesis (Ma et al., 2016). Myo-inositol is the precursor for the biosynthesis of many inositol derivatives including ascorbate acid, and is essential for plant growth and development (Gillaspy, 2011; Munnik and Nielsen, 2011). In plants, myo-inositol 1-phosphate synthase (MIPS1) catalyzes the rate-limiting step in myo-inositol synthesis (Donahue et al., 2010; Gillaspy, 2011; Munnik and Nielsen, 2011; Valluru and Van den Ende, 2011). Disruption of *MIPS1* results in reduction of myo-inositol biosynthesis, enhanced expression of plant defense genes, and a severe cell death phenotype (Meng et al., 2009; Donahue et al., 2010; Luo et al., 2011). The transcript abundance of *MIPS1* and *MIPS2*, and myo-inositol contents, are dramatically reduced in *fhy3* and *fhy3 far1* seedlings, which indicates that FHY3 and FAR1 positively regulate the transcription of *MIPS1/2* and the biosynthesis of myo-inositol (Ma et al., 2016). Further evidence indicates that FHY3 and FAR1 directly bind to the *MIPS1* and *MIPS2* promoters to activate their transcription and thus promote the biosynthesis of myo-inositol under light conditions. In addition, constitutive overexpression of *MIPS1* partially rescued inositol contents and the oxidative stress-induced cell death phenotype in the *fhy3 far1* mutant background. Therefore, FHY3 and FAR1 improve resistance to oxidative stress and suppress plant cell death also by positively regulating the biosynthesis of myo-inositol (Ma et al., 2016).

One surprising observation is that disruption of both *FRS7* and *FRS12* resulted in larger rosette leaves and plant size, and overexpression of *FRS7* or *FRS12* resulted in stunted growth under both long-day and short-day conditions, which indicate that FRS7 and FRS12 negatively regulate rosette leaf growth (Ritter et al., 2017). The molecular mechanism of how FRS7 and FRS12 negatively regulate rosette leaf growth is still unclear. It is worth noting that FRS7–FRS12 and FHY3–FAR1 have antagonistic roles in the growth of rosette leaves, since *fhy3 far1* and *frs7 frs12* adult plants display opposite growth phenotypes (Ma et al., 2016; Wang et al., 2016; Ritter et al., 2017).

### Abscisic Acid Signal Transduction and Stress Responses

Abscisic acid (ABA) plays multiple essential roles in plant growth and development including seed maturation, germination, gene expression, and stress responses (Wasilewska et al., 2008). Seed germination and seedling establishment are also regulated by light signals, yet how light and ABA synergistically regulate plant growth and development is still unclear (Lau and Deng, 2010). Recently, studies revealed that ABI5, a basic leucine zipper transcription factor, responds to both ABA and light signals (Finkelstein and Lynch, 2000; Tang et al., 2013).

Disruption of *FHY3* and *FAR1* reduced the ABA-dependent inhibition of seed germination, seedling greening, and root elongation. Compared with wild-type control plants, the mutant plants of *fhy3* are less sensitive to salinity and osmotic stresses. In addition, mutants with disruption function of *FHY3* and *FAR1* were less sensitive to ABA-induced stomatal closure, thus FHY3 and FAR1 are required for stomatal movement and drought response (Tang et al., 2013). Further investigation showed the transcript abundance of *ABI5* decreases in *fhy3*, *far1*, and *fhy3 far1* mutant seedlings, and overexpression of *ABI5* restores the *fhy3* mutant phenotype to wild-type levels, indicating that *ABI5* is regulated by FHY3 and FAR1. Therefore, FHY3 and FAR1 bind to the *ABI5* promoter and activate its transcription thus mediating ABA signal transduction and abiotic stress responses (Tang et al., 2013).

### Nutrient Absorption

Phosphorus (Pi) is an essential macronutrient and the limiting factor for plant growth, development, and metabolism. The MYB-type transcription factor PHOSPHATE STARVATION RESPONSE1 (PHR1) is crucial for the plant response to Pi deficiency (Rubio et al., 2001; Nilsson et al., 2007). Recent studies revealed that environmental light signals and ethylene in the soil work together to regulate *PHR1* transcription and ultimately the Pi starvation response (Liu Y. et al., 2017).

The light-signaling proteins FHY3 and FAR1 directly bind to the *PHR1* promoter to mediate the light-induced expression of *PHR1* and the Pi starvation response. Meanwhile, EIN3 and its closest homolog EIL1, two master transcription factors in the ethylene signal transduction pathway, also bind directly to the *PHR1* promoter to activate its expression and control the Pi starvation response. Disrupting both *FHY3* and *EIN3* significantly reduces Pi uptake in *fhy3 ein3* seedlings compared to wild-type control plants (Liu Y. et al., 2017). However, the bZIP transcription factor HY5 negatively regulates *PHR1* transcription. Thus, *PHR1* is positively regulated by FHY3 and FAR1 and negatively regulated by HY5 in response to light above-ground and ethylene stimuli in the soil. FHY3, FAR1, HY5, and EIN3 work together to regulate *PHR1* transcription and ultimately mediate the plant Pi starvation response (Liu Y. et al., 2017).

### The Protein Interaction Network of FRS-FRF Family Proteins

FHY3 has been found to physically interact with FAR1, FRS4, HY5, CCA1, LHY, PIF1, and EIN3 to regulate the transcription of various targets (**Table 1**; Wang and Deng, 2002; Li et al., 2010, 2011, 2016; Tang et al., 2012; Gao et al., 2013). Generally, FHY3 and FAR1 interact with each other to form homodimers or heterodimers and regulate the transcription of various target genes, but FHY3 plays the primary role in this regulation (Wang and Deng, 2002). CCA1, LHY, and PIF1 interact with FHY3 and suppress its transcriptional activation of *ELF4* and *HEMB1* (Li et al., 2011; Tang et al., 2012). HY5 interacts with FHY3 and coordinately promotes the expression of *ELF4* and *COP1* (Li et al., 2011; Huang et al., 2012), but suppresses the transcriptional activation of FHY3 on *FHY1* and *PHR1* (Li et al., 2010; Liu Y. et al., 2017). EIN3 interacts with FHY3 and coordinately promotes the expression of *PHR1* (Liu Y. et al., 2017). FRS4 interacts with FHY3 to promote the expression of *ARC5* (Gao et al., 2013), and FRS3 interacts with JAZ3 and ZML2, as shown by a proteome-wide protein–protein interaction network analysis (Arabidopsis Interactome Mapping Consortium, 2011). In addition, tandem affinity purification-mass spectrometry showed that FRS7, HON4 (Histone-like protein 4), and AHL14 (AT-Hook motif nuclear localized protein 14) co-purified with FRS12, and that FRS7–FRS12 acts as part of a transcriptional repressor complex (Ritter et al., 2017). Many proteins have been found to interact with FHY3 or other FRS family proteins, and most of them are transcription factors (**Table 1**). It remains to be determined how different FRS family members interact with other transcriptional regulators, such as regulators of histone modification or other signal transduction proteins, to coordinate the regulation of the transcription of various genes.

### FRS-FKF Family Proteins in Other Plant Species

Over 1000 putative FRS homologs have been predicted in diverse higher plant species, which indicates that FRS-FRF family proteins are evolutionarily conserved in angiosperms (Lin et al., 2007; Joly-Lopez et al., 2016). Beside *Arabidopsis thaliana*, the physiological functions of FRS family members have not been investigated in other higher plant groups. However, recent studies have suggested that putative FRS members are the candidate genes for *Panicle and Spikelet Degeneration* (*PSD*) gene in rice (Zhang et al., 2015), and a photoperiod-dependent flowering time regulator in wheat (Kiseleva et al., 2017). In addition, genomic sequencing and population genomic analysis of silver birch (*Betula pendula*) also demonstrated that a putative FRS10 member might be the candidate gene correlated with the adaptation to environment (Salojarvi et al., 2017). Thus, it would be interesting to investigate the physiological function of FRS-FRF family protein in other species. Indeed, in *Aspergillus nidulans*, VipA contains a FAR1-like DNA-binding domain and modulates light-regulated heme biosynthesis through direct association with the *hemB* promoter, indicating that FRS family proteins also function in filamentous fungi (Rohrig et al., 2017).

## Concluding Remarks and Future Perspectives

FHY3, FAR1, and other FRS family members regulate various cellular processes by transcriptionally activating or repressing the expression of diverse target genes. ChIP-seq, DAP-seq, and tandem chromatin affinity purification-sequencing have identified thousands of different target genes that are subject to transcriptional regulation by FHY3, FAR1, or FRS7; however, only a few of them have been functionally characterized so far (Ouyang et al., 2011; Li et al., 2016; O’Malley et al., 2016; Ritter et al., 2017). Therefore, the FRS family proteins might have roles that are more important than was originally thought. In addition, FHY3–FAR1 and FRS7–FRS12 recognize distinct *cis*-elements in the promoters of their target genes (Lin et al., 2007; Ouyang et al., 2011; Li et al., 2016; O’Malley et al., 2016; Ritter et al., 2017), suggesting that different FRS family members might have different DNA-binding activities and gene activation features. Further exploring the physiological roles of the FRS family proteins and their gene targets will expand our understanding of how different FRS family proteins regulate plant growth and development. Therefore, to characterize the new physiological functions of various FRS-FRF family proteins is one of the important future directions in plant biology research.

FHY3 and FAR1 primarily activate the transcription of light-induced target genes, while FHY3 also represses another set of genes through an unknown mechanism (Ouyang et al., 2011). The precise molecular mechanism of how FHY3 or other FRS members activate or repress the transcription of various targets has remained elusive, but other transcriptional regulators such as Polycomb Repressive Complex 2 or the Mediator complex might be involved in these processes. However, when and why FHY3 (or other FRS family proteins) act as either activators or repressors in different organs or development stages will need to be determined in the future.

FRS family proteins are derived from transposases. Numerous studies have demonstrated that transposable elements and selfish elements are powerful contributors to genome evolution and diversity in angiosperms (Oliver et al., 2013). Although FHY3 and FAR1 probably did not retain transposase activity in *Arabidopsis*, it remains to be explored whether the FRS family proteins in other plants have retained transposase activity or have developed novel activity as transcription factors. FHY3 and FAR1 are the best characterized plant transcription factors that were derived from transposons. How other transcription factors were derived from transposons and diversified into the key transcriptional regulators they are today is worthy of further investigation.

## Author Contributions

LM and GL drafted and approved the final version of the manuscript.

## Conflict of Interest Statement

The authors declare that the research was conducted in the absence of any commercial or financial relationships that could be construed as a potential conflict of interest.
